# xCas9 expands the scope of genome editing with reduced efficiency in rice

**DOI:** 10.1111/pbi.13053

**Published:** 2019-01-02

**Authors:** Junjie Wang, Xiangbing Meng, Xixun Hu, Tingting Sun, Jiayang Li, Kejian Wang, Hong Yu

**Affiliations:** ^1^ State Key Laboratory of Rice Biology China National Rice Research Institute Chinese Academy of Agricultural Sciences Hangzhou China; ^2^ State Key Laboratory of Plant Genomics and National Center for Plant Gene Research (Beijing) Institute of Genetics and Developmental Biology The Innovative Academy of Seed Design Chinese Academy of Sciences Beijing China; ^3^ University of Chinese Academy of Sciences Beijing China

**Keywords:** xCas9, genome editing, rice

Dear Editor,

CRISPR‐Cas9 systems have been widely used in animals and plants for genome editing, epigenetic modification, etc. (Cong *et al*., 2013; Hilton *et al*., 2015). However, the accurate recognition of target sites of CRISPR/Cas9 systems depends on the single‐guide RNA (sgRNA) corresponding to each target site and protospacer adjacent motifs (PAMs) around these sites (Anders *et al*., 2014). Cas9‐sgRNA complexes will directly eject the DNA template if no PAMs are recognized before base pairing between target sites and sgRNA (Sternberg *et al*., 2014). Therefore, PAM recognition is an essential step for the function of Cas9‐sgRNA complexes. The common *Streptococcus pyogenes* Cas9 (*Sp*Cas9) recognizes canonical NGG PAM, which restricts the editable range of the rice genome. In an effort to overcome this limitation, several studies have reported that other Cas effectors (Cpf1 for AT‐rich PAMs) and engineered Cas9 variants (VQR for NGA PAMs and VRER for NGCG PAMs) could be employed with other PAMs for rice genome editing (Hu *et al*., 2016; Zetsche *et al*., 2015). In addition, another study showed that *Sp*Cas9 could robustly recognize NAG PAMs and cleave target sites in rice genome (Meng *et al*., 2018). Although several tools have been developed for genome editing, versatile nucleases recognizing various PAMs are still largely required to expand the genome editing toolbox. Recently, xCas9, a Cas9 variant that recognizes most types of PAM reported in mammalian cells, including NG, GAA and GAT, was developed (Hu *et al*., 2018). However, the efficiency of this system in other species has not been reported yet. Here, we generated two versions of efficient xCas9 variants to expand the scope of genome editing in rice.

To generate Cas9 variants, we modified rice codon‐optimized *Sp*Cas9 (Wang *et al*., 2015). Two types of xCas9 variants, E108G/S217A/A262T/S409I/E480K/E543D/M694I/E1219V and A262T/R324L/S409I/E480K/E543D/M694I/E1219V (Figure 1a), were generated by PCR site‐directed mutagenesis; these variants are hereafter referred to as xCas9 3.6 and xCas9 3.7, respectively (Hu *et al*., 2018). Because xCas9 recognizes GAT, GAA and NG PAMs in mammalian cells (Hu *et al*., 2018), the plausibility of genome editing using xCas9 variants in rice was tested by designing 18 target sites harbouring GAT, GAA and NG PAMs. Three rice endogenous genes, *MONOCULM1* (*MOC1*), *DWARF14* (*D14*) and *PHYTOENE DESATURASE* (*PDS*), were involved with each PAM. Independent sgRNAs of each PAM were assembled under U3 promoters and final binary vectors, which included three cassettes of different sgRNAs and one cassette of *Cas9* (wild type or variants), using the isocaudomer ligation method (Wang *et al*., 2015). The callus was selected by hygromycin B for further detection after *Agrobacterium*‐mediated transformation. To determine whether Cas9 variants induced mutations in rice calli, the target sites were amplified and analysed using previously developed high‐throughput tracking of mutations (Hi‐TOM) platform (Liu *et al*., 2018). The results showed that at non‐canonical GAA PAM sites, the editing efficiency of xCas9 3.7 (2.08%–12.5%) was higher than that of xCas9 3.6 (0%–4.17%), whereas no mutations were detected at GAA PAM sites with *Sp*Cas9. At GAT PAM sites, no mutations were detected with xCas9 3.6, xCas9 3.7 and *Sp*Cas9. Although xCas9 3.6 (2.08%–8.03%) and xCas9 3.7 (4.17%–18.37%) displayed the ability to edit AGA, TGT and CGC PAM sites, its efficiency at canonical GGG PAM site (4.17%–10.42%) was significantly reduced compared with that of *Sp*Cas9 (75.00%–77.08%; Figure 1b). These results indicate that xCas9 could expand the scope of genome editing with some reported non‐canonical PAMs in rice.

**Figure 1:**
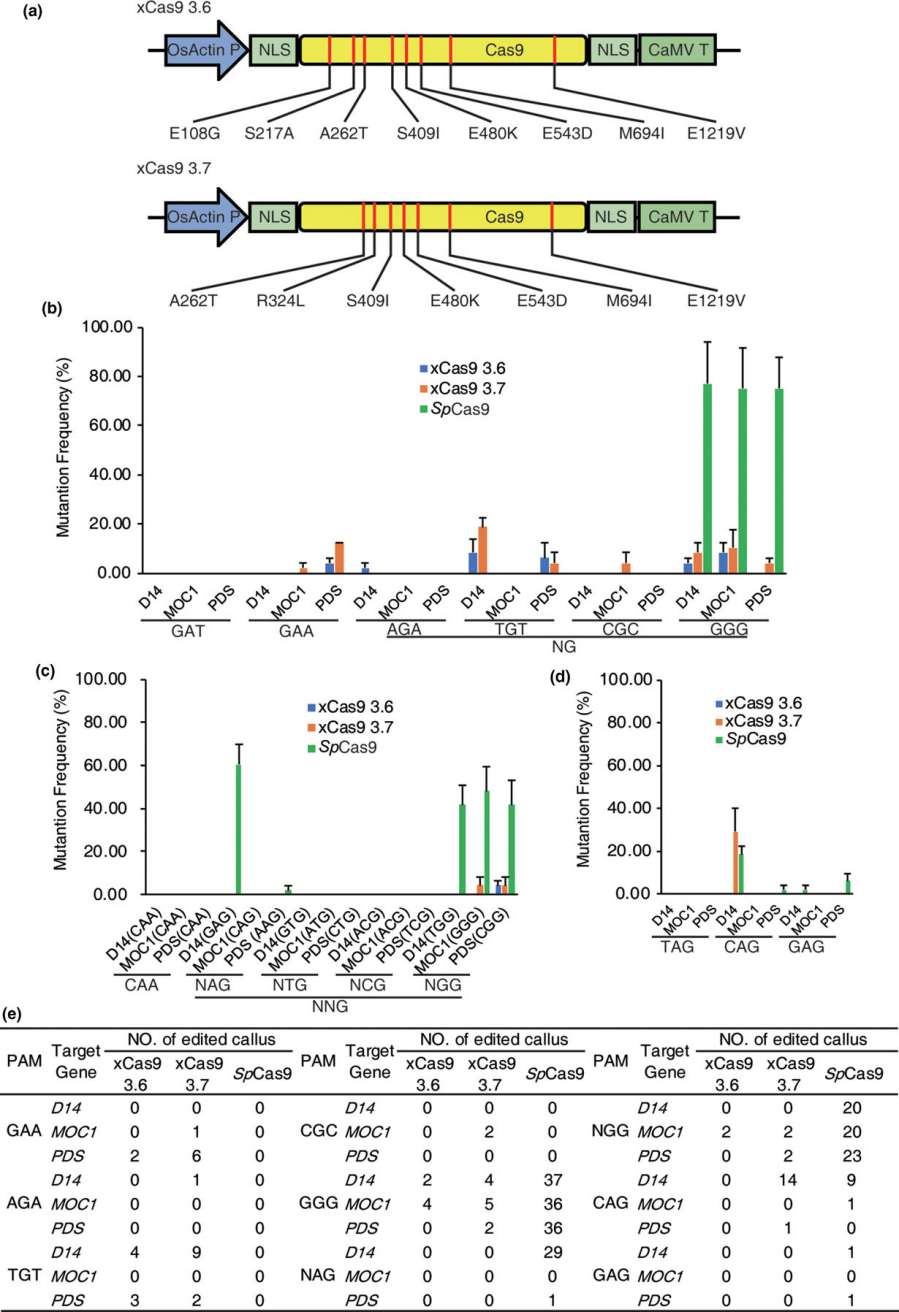
Genome Editing in Rice Using xCas9 3.6 and 3.7 Variants. (a) Schematic illustration of the generation of xCas9 3.6 and 3.7 variants. NLSs: nuclear localization signals, CaMV T: cauliflower mosaic virus 35S terminator, OsActin P: *OsActin* promoter. Red lines represent differences in amino acid positions compared with *Sp*Cas9. (b) Efficiency of genome editing using reportedly efficient protospacer adjacent motifs in three different endogenous rice genes. Error bars represent mean ± SEM (*n* = 3 independent replicates, with each independent replicate containing 16 transformed calli). (c) Efficiency of genome editing at CAA and NNG PAM sites in three different endogenous rice genes. Error bars represent mean ± SEM (*n* = 3 independent replicates, with each independent replicate containing 16 transformed calli). (d) Efficiency of genome editing at NAG PAM sites in three different endogenous rice genes. Error bars represent mean ± SEM (*n* = 3 independent replicates, with each independent replicate containing 16 transformed calli). (e) Summary of edited calli as tested by Hi‐TOM analysis. Hi‐TOM analysis filter threshold is set as >5%. Total number of detected calli is 48, (three independent replicates, with each independent replicate containing 16 transformed calli). *MOC1*:* MONOCULM1*,* D14*:* DWARF14* and *PDS*:* PHYTOENE DESATURASE* [Colour figure can be viewed at wileyonlinelibrary.com]

In addition, we performed genome editing with another two PAMs, CAA and NNG, which was shown to be efficient in a PAM depletion assay conducted in a previous study (Hu *et al*., 2018). Overall, 15 target sites were designed for CAA and NNG (including NAG, NTG, NCG and NGG) PAMs, in which the efficiency of each PAM was assessed at three target sites. For each target site, 48 transgenic rice calli were generated and examined using Hi‐TOM. No mutations were detected around CAA PAM sites in lines of xCas9 variants and *Sp*Cas9. Among the four NNG PAMs assessed, xCas9 3.6 and 3.7 exhibited limited ability (4.17%) to edit three NGG PAM sites (Figure 1c). In addition to NGG PAM sites, *Sp*Cas9 also effectively introduced mutations at NAG PAM sites (2.08%–60.42%), which is consistent with previous report (Meng *et al*., 2018).

To further determine the ability of xCas9 variants to edit NAG PAM sites, nine target sites in *MOC1*,* D14* and *PDS* harbouring three NAG PAMs (TAG, CAG and GAG PAM) were designed. The result showed that neither the xCas9 variants nor *Sp*Cas9 exhibited any gene editing ability at TAG PAM sites. However, at the CAG PAM site (targeting *D14*), the efficiency of the xCas9 3.7 variants (29.17%) was comparable to that of *Sp*Cas9 (2.08%–18.75%). At GAG PAM site, mutations were detected with *Sp*Cas9 (2.08%–6.25%) but not with xCas9 variants (Figure 1d). Collectively, these results show that for NAG PAM sites, xCas9 3.7 shows a lower activity compared to *Sp*Cas9; xCas9 3.6 was inefficient at NAG PAM sites in our study.

In this study, we generated two xCas9 variants, xCas9 3.6 and xCas9 3.7, based on the rice codon‐optimized sequence of *Sp*Cas9 by modifying different *Sp*Cas9 amino acids. By comparing the mutation rates caused by xCas9 variants and *Sp*Cas9 in 63 target sites harbouring 21 PAMs, we showed that xCas9 variants can recognize a variety of PAMs including GAA and NG, which greatly expands the scope of genome editing in rice. However, the efficiency at those sites was relatively low. By analysing efficiency at NGG PAM and GGG PAM sites, we found that the efficiency of xCas9 was significantly less than that of *Sp*Cas9 for canonical NGG PAMs (Figure 1e). We also tested xCas9 variants with other PAMs, such as TAH, CAY, GTA and GAC PAMs, and revealed that none of them could be recognized by xCas9 variants in rice. Moreover, xCas9 3.7 could recognize NAG PAM, a recently reported highly efficient PAM in rice, at a relatively low level. Compared with mammalian cells, the efficiency of xCas9 in rice is much lower, which may be due to the differences in temperature and intracellular environment. Next, further work such as optimizing the conditions of genome editing are required to improve the working efficiency of xCas9 in plants.

Overall, xCas9 3.7 performed better than xCas9 3.6 for genome editing in rice, showing the potential of xCas9 variants to become versatile tools which will expand the scope of genome editing in rice.

## Competing Financial Interests

The authors declare no conflict of interest.

## Author contributions

K.W., H.Y. and J.L. designed the studies. J.W., X.M. and X.H. performed the experiments; T.S. conducted the bioinformatic analyses. K.W., H.Y., J.L., J.W., X.M. and X.H. wrote the manuscript.
